# Anti-SARS-CoV-2 Antibodies Testing in Recipients of COVID-19 Vaccination: Why, When, and How?

**DOI:** 10.3390/diagnostics11060941

**Published:** 2021-05-25

**Authors:** Giuseppe Lippi, Brandon Michael Henry, Mario Plebani

**Affiliations:** 1 Section of Clinical Biochemistry, University of Verona, 37126 Verona, Italy; 2 Cincinnati Children’s Hospital Medical Center, The Heart Institute, Cincinnati, OH 3333, USA; brandon.henry@cchmc.org; 3 Department of Medicine-DIMED, Medical School, University of Padova, 37126 Verona, Italy; mario.plebani@unipd.it; 4 Department of Laboratory Medicine, University-Hospital of Padova, 37126 Verona, Italy

**Keywords:** SARS-CoV-2, COVID-19, vaccination, monitoring, immunoassays

## Abstract

Although universal vaccination is one of the most important healthcare strategies for limiting SARS-CoV-2 (severe acute respiratory syndrome coronavirus 2) circulation and averting the huge number of hospitalizations and deaths due to coronavirus disease 2019 (COVID-19), significant inter-individual variability of COVID-19 vaccines’ efficacies has been described, mostly due to heterogeneous immune response in recipients. This opinion paper hence aims to discuss aspects related to the opportunity of monitoring anti-SARS-CoV-2 antibodies before and after COVID-19 vaccination, highlighting the pros and cons of this strategy. In summary, the advantages of anti-SARS-CoV-2 antibodies’ testing in recipients of COVID-19 vaccination encompass an assessment of baseline seroprevalence of SARS-CoV-2 infection in non-vaccinated individuals; early identification of low or non-responders to COVID-19 vaccination; and timely detection of faster decay of anti-SARS-CoV-2 antibody levels. In contrast, potential drawbacks to date include an unproven equivalence between anti-SARS-CoV-2 antibody titer, neutralizing activity, and vaccine efficiency; the lack of cost-effective analyses of different testing strategies; the enormous volume of blood drawings and increase of laboratory workload that would be needed to support universal anti-SARS-CoV-2 antibodies testing. A potential solution entails the identification of cohorts to be prioritized for testing, including those at higher risk of being infected by variants of concern, those at higher risk of unfavorable disease progression, and subjects in whom vaccine immunogenicity may be expectedly lower and/or shorter.

## 1. Introduction

Coronavirus disease 2019 (COVID-19), which is a severe infectious disease that was initially reported as an “unknown cause of pneumonia” in Wuhan City, China, on 31 December 2019, has now reached pandemic proportions, already causing over 3 million deaths worldwide [1]. Reliable evidence now suggests that the establishment of physical preventive measures, such as lockdowns, social distancing, widespread usage of face masks, and hand hygiene are only partially effective for preventing or limiting the tragic impact of COVID-19 on human health, society, and the economy [2]. Therefore, universal vaccination now appears to be the most important tool for limiting viral circulation, but also for averting the huge number of hospital admissions and deaths that are associated with SARS-CoV-2 (severe acute respiratory syndrome coronavirus 2) infections [3,4].

The current armamentarium of COVID-19 vaccines encompasses different options, as represented by inactivated virus, viral proteins (e.g., SARS-CoV-2 spike protein), and DNA- and mRNA-based vaccines [5,6], the last generation of which entails lipid-based mRNA nanoparticles vaccines (mRNA-LNPs) [7]. The data published to date attest that the different vaccines have a considerably high efficacy (i.e., between 85 and 100%) in preventing the risk of developing severe or critical forms of COVID-19 illness caused by the prototype Wuhan strain, whilst displaying relatively lower clinical efficiency (i.e., between 50 and 80%) in patients with infections caused by new SARS-CoV-2 variants and a similarly limited protection against any type of SARS-CoV-2 infection [8]. This would hence imply that some forms of monitoring of vaccine efficacy would be advisable for predicting individual and community immunogenicity and the consequent efficacy in all potential recipients [9]. Therefore, the present opinion paper aims to discuss some aspects related to the opportunity of monitoring anti-SARS-CoV-2 antibodies before and after COVID-19 vaccinations.

## 2. Why?

The baseline serological monitoring now appears as a valid option for guiding vaccine administration for at least three major reasons, i.e., assessing the baseline seroprevalence of SARS-CoV-2 infection in non-vaccinated individuals, evaluating the immune response in both anti-SARS-CoV-2 seronegative or seropositive subjects at baseline, and eventually reducing the risk of developing adverse events after a COVID-19 vaccination in patients with previous SARS-CoV-2 infection, whether symptomatic or asymptomatic. 

While the first point is easily intuitive, the individual humoral response to COVID-19 vaccination remains largely unpredictable. For example, the two-dose complete cycle of mRNA COVID-19 vaccination seems to generate a lower relative increase of anti-SARS-CoV-2-neutralizing antibodies in people with a previous SARS-CoV-2 infection than in those who were not infected by the virus. This seems mostly attributable to the fact that most people with a previous SARS-CoV-2 infection have already developed a straightforward humoral, cellular, and memory immune response, and thus a further increase of anti-SARS-CoV-2 antibodies after the second vaccine dose is modest in subjects who have strongly responded to the first [10,11,12,13]. Notably, a significant correlation has been observed between pre-vaccination anti-SARS-CoV-2 antibodies titer and Pfizer BNT162b2 vaccine immunogenicity, whereby the anti-SARS-CoV-2 RBD (receptor binding domain) total antibodies response seems to be reduced (up to nearly eightfold) in patients with a baseline antibody level in the upper quartile [11]. Therefore, with 50% (or even more) of all SARS-CoV-2 infections remaining completely asymptomatic up to negativization of molecular testing [14,15], the organization of pre-vaccination serological surveys represents a reliable mean for unmasking all SARS-CoV-2 seropositive subjects in whom vaccine administration may be personalized, adapted, or even delayed based on their actual anti-SARS-CoV-2 antibodies status [16]. This would also enable the optimization of vaccine administration to those who may benefit most (i.e., baseline anti-SARS-CoV-2 seronegative subjects), thus contributing to at least partially overcoming the current vaccines’ manufacturing bottleneck, which is causing a worldwide shortage of doses, and thus perpetuating new outbreaks and the potential for emergence of new mutant strains.

Some recent population studies conducted on baseline anti-SARS-CoV-2 seronegative subjects revealed that immunogenicity after mRNA vaccination differed widely between recipients, with variations of neutralizing antibodies’ levels extending between 1 and 2 orders of magnitude [10,11,12]. In particular, Salvagno et al. recently highlighted that women and subjects aged <60 years had 20% and 30% higher values of anti-SARS-CoV-2 RBD total antibodies after a complete Pfizer BNT162b2 vaccine cycle compared to men and the elderly, respectively. Overall, older men had a nearly 50% lower anti-SARS-CoV-2 RBD total antibody response compared to young women [11]. Similar evidence was garnered in other studies, whereby lower vaccine immunogenicity was also reported in older people and/or in males [17,18,19,20,21]. Given the increased risk for poor COVID-19 progression and outcomes associated with advanced age and male sex, these groups may be especially vulnerable in the setting of a diminished immune response [22]. Notably, concern that obese and/or overweight persons may suffer attenuated immunogenicity of COVID-19 vaccination was also recently expressed. In particular, Pellini et al. published preliminary data in recipients of the Pfizer BNT162b2 vaccine, showing that the levels of anti-SARS-CoV-2 spike IgG antibodies elicited by vaccination were between 50–60% lower in pre-obese and obese individuals [21]. Obesity is also a major risk factor for severe COVID-19 and is associated with higher mortality [23]. 

Vaccine-elicited immunogenicity was also found to be considerably attenuated in some specific populations of patients. Deepak et al. observed a threefold lower generation of anti-SARS-CoV-2 spike IgG and neutralizing antibodies in patients with chronic inflammatory diseases undergoing immunosuppressive treatment compared to immunocompetent controls [24]. Similar evidence was collected from other studies in patients receiving various immunosuppressive agents after transplantation [18,25,26,27,28] or treatment for chronic inflammatory conditions [29], but also in patients with different forms of cancer [30], especially in those with hematological malignancies [31,32], as well as in subjects with end-stage renal disease [33].

The possible occurrence of side effects is the third important aspect underlying the importance of monitoring the anti-SARS-CoV-2 response in recipients of COVID-19 vaccines. Evidence has accumulated showing that mRNA vaccination may be more frequently associated with adverse events in SARS-CoV-2 seropositive patients than in naïve individuals. Compared with baseline seronegative subjects, Krammer reported higher frequencies of fatigue (44% vs. 25%), headache (36% vs. 17%), muscle pain (28% vs. 8%), and fever (22% vs. 4%) after the first vaccine dose in baseline seropositive subjects [13]. Manni et al. also showed that self-reported systemic adverse events were higher in SARS-CoV-2 seropositive patients than in seronegative individuals after both the first (35% vs. 12%) and second (38% vs. 20%) mRNA BNT162b2 vaccine doses [34]. Therefore, the timely detection of baseline SARS-CoV-2 seropositive subjects displaying an efficient antibody level may avert the risk of developing adverse events due to unwarranted vaccine administration (i.e., in the presence of appropriately high neutralizing antibody titers).

Concerns were expressed that vaccine efficacy may be significantly lowered by the emergence of the so-called variants of concern (VOCs), such as B.1.1.7 (i.e., the “U.K. variant”), B.1.351 (i.e., the “South-African variant”), P.1 (i.e., the “Brazilian variant”), B.1.617 (i.e., the “Indian variant”), and B.1.427/B.1.429 (i.e., the “USA-California variants”) [35,36]. All these VOCs, which seem to have independently emerged at different latitudes from convergent selection pressure, tend to share some important mutations, such as L452R, E484K, N501Y, and D614G, which either increase their binding affinity to host receptors (especially to angiotensin-converting enzyme 2; ACE2), or may variably alter their immunogenicity, such that they are less efficiently counteracted by humoral and cellular immune responses that are developed after natural infection with another SARS-CoV-2 strain or after administration of vaccines based on the prototype Wuhan strain [37]. In a seminal article published by Hoffmann et al., it was recently reported that the neutralizing potency of antibodies elicited after SARS-CoV-2 infection or COVID-19 vaccination would be decreased by approximately 1.7-, 5.0-, and 7.9-fold versus the B.1.1.7, P.1, and B.1.351 lineages, respectively [38]. Similar data were published by others regarding the recipients of different vaccine formulations, such as Pfizer BNT162b2 or Oxford-AstraZeneca ChAdOx1 nCoV-19 [39], Pfizer BNT162b2 or Moderna mRNA-1273 [40], or Sputnik Gam-COVID-Vac [41]. This biological evidence may then translate into a tangible clinical risk, as was recently reported by some preliminary prospective studies. For example, Shinde et al. followed-up over 4000 subjects that were randomized to receive at least one dose of Novavax NVX-CoV2373 vaccine (i.e., recombinant spike protein with Matrix-M1 adjuvant) or a placebo [42], and found that the vaccine efficacy versus the B.1.351 lineage was only 51.0% (95% CI, −0.6 to 76.2%). According to the data garnered so far, the decay of the neutralization potential of anti-SARS-CoV-2 antibodies against the currently known VOCs compared to the prototype SARS-CoV-2 strain is summarized in Table 1.

Liu Y et al. observed a lower but still robust neutralization of the B.1.351 spike virus in BNT162b2-elicited serum, emphasizing that T-cell immunity may also be involved in the protection, and that real-world evidence collected in regions where SARS-CoV-2 variants are circulating is urgently needed [43]. In another study from Qatar, which involved nearly 400,000 individuals, the authors found that the administration of a complete cycle of Pfizer BNT162b2 vaccine displayed 17% lower efficacy toward preventing infection from the B.1.351 variant compared to the prototype Wuhan strain (i.e., 75% vs. 92%) [44]. Further studies are needed to clarify whether mutations outside of the RBD (e.g., polymorphisms in the NTD at positions 144, 152, 156, 157) may have a synergistic effect toward reducing the neutralization potential of anti-SARS-CoV-2 antibodies.

Taken together, these findings provide evidence in favor of systematic monitoring of anti-SARS-CoV-2 antibody response both before and after COVID-19 vaccination, allowing us to also identify some demographical and clinical factors, the presence of which may also allow for prioritizing post-vaccination serological assessment (Table 2).

## 3. When?

The second essential aspect of the serological assessment of recipients of COVID-19 vaccinations is establishing when blood should be drawn and testing conducted. Although the relatively recent introduction of COVID-19 vaccines has not yet allowed for garnering comprehensive information on the persistence of immune responses, some important evidence has become available. Doria-Rose et al. described the neutralizing antibodies’ responses in 33 healthy adults who received the Moderna mRNA1273 vaccine [45], where they reported a steady decay rate over time, with a half-life between 109 and 119 days for anti-SARS-CoV-2 RBD antibodies and between 69 and 173 days for anti-SARS-CoV-2 neutralizing antibodies, respectively. These results overlap with those reported in patients with natural SARS-CoV-2 infection, in whom the half-life of anti-SARS-CoV-2 RBD IgG antibodies was found to be ~110 days [46]. In a separate investigation, Ketas et al. monitored the immunogenicity of COVID-19 vaccination over time in 45 healthy recipients, in whom a sustained response of all anti-SARS-CoV-2 RBD antibodies classes was still evident 3 months after receiving a complete set of vaccinations with either Pfizer BNT162b2 or Moderna mRNA-1273 mRNA vaccines [47]. 

**Figure 1:**
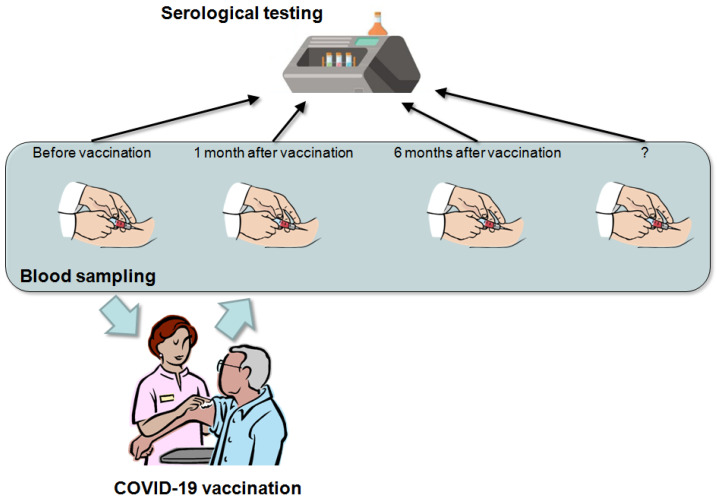
Suggested time points for anti-SARS-CoV-2 antibodies’ titration in recipients of COVID-19 vaccinations, encompassing a baseline assessment, followed by antibody level monitoring at least 1 and 6 months after the last vaccine dose.

Therefore, although additional information is necessary to establish medium- and long-term plans for anti-SARS-CoV-2 antibodies’ titration after COVID-19 vaccinations, the current evidence would suggest that the initial time points may entail a baseline assessment followed by serial measurements at 1 and 6 months after the last vaccine dose (for vaccines encompassing more than one dose administration) (Figure 1). 

The 1-month measurement would thus enable a timely identification of low- or non-responders to the vaccination, whilst the 6-month assessment may allow for recognizing vaccine recipients displaying a faster antibody decay rate. In both cases, consideration could then be made for strengthening preventive measures or even administering vaccine boosters [48]. Additional time points for drawing blood and anti-SARS-CoV-2 serological testing will then be defined as evidence on the kinetics of the humoral (and cellular) immune response accumulates over the coming months.

## 4. How?

There is now solid evidence that the neutralization activity of post-infection or post-vaccination serum mostly resides in the presence of antibodies targeting the SARS-CoV-2 spike protein and or its RBD. An elegant study that was recently published by Voss and colleagues demonstrated that up to 84% of all neutralizing antibodies target sequences located outside the RBD and that those binding the N-terminal domain (NTD) of the S1 subunit of the SARS-CoV-2 spike protein were highly protective in a model of lethal viral challenge [49]. This would actually mean that the selected immunoassay shall be preferably based on detection of antibodies targeting the SARS-CoV-2 trimeric spike glycoprotein conformation, its S1(/S2) subunit, and its RBD, since these will reflect the greatest burden of neutralizing potential. Needless to say, the use of these immunoassays would be mandatory in recipients of mRNA- or DNA-based vaccines encoding for SARS-CoV-2 spike protein, as well as in people receiving those based on direct administration of a recombinant form of the SARS-CoV-2 spike protein, whereby only antibodies against these protein moieties will be elicited in baseline seronegative recipients. No clear evidence has been provided on which anti-SARS-CoV-2 antibodies assessment (total or IgG) would be preferable for the purpose of anti-SARS-CoV-2 antibodies’ testing in recipients of COVID-19 vaccinations, such that definitive indications on this matter cannot be made, which is a concept that was also noted by the Task Force on COVID-19 of the International Federation of Clinical Chemistry and Laboratory Medicine (IFCC) [50]. That said, the measurement of anti-SARS-CoV-2 IgM antibodies in this setting was ruled out (due to their questionable protective role, modest increase, and fast decay) [51,52], while consideration should be given to anti-SARS-CoV-2 IgA assessment, since this antibody class may reflect the efficiency of mucosal protection against SARS-CoV-2 infection [53].

Another important consideration is the choice between qualitative, semi-quantitative, or quantitative serological anti-SARS-CoV-2 immunoassays. As previously discussed, an obvious preference should be given to automated techniques (for supporting larger volumes of testing) that provide an accurate quantitative measure of the antibody titer since this would enable defining the pre-vaccination “baseline” (especially in baseline seropositive individuals), as well as allowing for straightforward and accurate monitoring of anti-SARS-CoV-2 antibodies’ decay over time [54]. Regarding the choice between the different available techniques, the issue of using different methodological approaches could further contribute toward challenging standardization and/or harmonization initiatives, wherein different anti-SARS-CoV-2 immunoassays detect different immunoglobulins targeting different viral epitopes and are characterized by heterogeneous detection limits, different cut-offs, and dissimilar ranges of antibody concentrations. This shall hence be seen as a tangible hurdle in setting a universal approach for "personalized vaccination.” Although an international standard was recently introduced (i.e., the WHO 20/136 standard, which enables reporting test results in binding antibody units per mL; BAU/mL) [55], acceptable harmonization of the many commercially available immunoassays is hence still expected to present many challenges [56]. Therefore, the use of an identical immunoassay seems highly advisable for the purpose of longitudinal monitoring of the anti-SARS-CoV-2 antibodies’ kinetics in vaccine recipients. Consideration could also be made for assessing IgG2a and IgG1 for detecting type 1 T helper (TH1) and type 2 T helper (TH2) immune responses, respectively.

Finally, with respect to the preferable setting of serological testing, conventional clinical laboratories provide the best guarantee of safety and accuracy, as traditional laboratory instrumentation has higher diagnostic performance (in terms of accuracy, precision, and throughput) compared to rapid and/or point-of-care tests, laboratory professionals can provide better counseling regarding the clinical implications of test results, and the produced data that can be stored in the laboratory information system (LIS), where they are later available for the purpose of longitudinal monitoring (Table 3) [57].

## 5. Potential Drawbacks

While anti-SARS-CoV-2 antibody testing in recipients of COVID-19 vaccinations has many advantages, as earlier discussed, some possible drawbacks should be highlighted, as summarized in Table 4. 

The first such caveat is that the equivalence between the anti-SARS-CoV-2 antibody levels and vaccine efficacy is not so straightforward, since the role played by cellular immunity and memory B cells cannot be ascertained only through serological testing [58]. Therefore, although cases of SARS-CoV-2 re-infection due to waiving of humoral immunity, VOCs, or both are increasingly reported [59], more research is urgently needed to define (i) the individual protective threshold level of anti-SARS-CoV-2 antibodies below which the humoral defense against different SARS-CoV-2 variants is more likely to fail [60], (ii) whether the memory B cells primed by previous SARS-CoV-2 infection or COVID-19 vaccination can be rapidly reactivated and will be capable of generating an efficient anti-SARS-CoV-2 antibody level [61,62], and (iii) the role of cell-mediated immunity in protecting from SARS-CoV-2 infection and especially in averting the risk of developing severe or critical forms of the disease. Besides the still uncertain relationship with vaccine efficacy, evidence was also published showing that the different commercially available anti-SARS-CoV-2 immunoassays display variable agreement with neutralization tests [63].

The second important drawback is that the cost-effectiveness of a widespread serosurveillance strategy after COVID-19 vaccinations is still unknown. The range of costs for a single laboratory-based anti-SARS-CoV-2 test is rather ample (most typically between 1 and 5 USD), though prices largely differ from one manufacturer to another, but can also vary for the same test across different geographies or healthcare settings. Therefore, a universal approach that could provide a thoughtful answer to this question is almost unfeasible. Local simulation studies should be planned, where the overall costs could be calculated based on available economical resources, type of COVID-19 vaccine, number of total (serial) samplings, type of anti-SARS-CoV-2 immunoassay, cost of staff and sample delivery, and so forth.

The volume of potential tests and their logistic impact are other important issues, since the number of people who may need/wish to have their anti-SARS-CoV-2 antibodies’ level monitored over time is unlikely to be manageable by any healthcare system worldwide. This would entail the establishment of blood drawing facilities with an adequate workforce to avoid saturating conventional phlebotomy centers, more or less like what was done with the creation of dedicated COVID-19 diagnostic and vaccination centers [64,65]. Clinical laboratories should also be prepared to withstand a consistent increase of their serological workload in circumstances of extensive population testing.

## 6. Conclusions

It is undeniable that anti-SARS-CoV-2 antibodies’ testing in recipients of COVID-19 vaccinations offers many clinical advantages, but may also bring important biological, economical, and logistical challenges. The evidence garnered so far would not allow for drawing definitive conclusions on this matter, though we hope that our analysis may persuade policymakers, healthcare administrators, clinicians, and even laboratory professionals to establish multidisciplinary teams that are aimed at defining the local cost-effectiveness of post-COVID-19 vaccination serosurveillance campaigns. A possible good sense solution encompasses the identification of cohorts to be prioritized for testing, e.g., those at higher risk of being infected by VOCs, or those in whom vaccine immunogenicity appears lower and/or shorter, such as older men, subjects with a high body mass index or undergoing immunosuppressive treatments, and patients with cancer or severe impairment of renal function, who are also at higher risk of developing more severe forms of COVID-19 illness (Table 2) [66]. Notably, the recent claim made by the WHO stating that international COVID-19 trials shall be restarted with more focus on immune response provides significant support to targeted anti-SARS-CoV-2 antibodies testing for assessing the effectiveness of existing treatments, thus including vaccinations [67].

## Figures and Tables

**Table 1:** Decay of the neutralization potential of anti-SARS-CoV-2 antibodies against the currently known variants of concern (VOCs) compared to the prototype SARS-CoV-2 strain.

Variants of Concern	B.1.1.7	B.1.351	P.1	B.1.617	B.1.427/B.1.429
Original emergence	U.K.	South Africa	Brazil	India	USA (California)
Decay of neutralization potential *	~1.7-fold	~7.9-fold	~5.0-fold	~6.8-fold	~3.6-fold

* Compared to the prototype SARS-CoV-2 strain.

**Table 2:** Demographical and clinical factors associated with a lower COVID-19 vaccine immune response.

1. Male sex;
2. Older age (i.e., >65 years);
3. High body mass index (i.e., ≥25 kg/m^2^);
4. Immunosuppressive treatments;
5. Cancer (especially hematologic malignancies);
6. End-stage renal disease/dialysis;
7. Endemic appearance of novel variants of concern (VOCs).

**Table 3:** Technical and logistic consideration for serological testing in recipients of COVID-19 vaccination.

1. Prefer immunoassays detecting antibodies targeting the SARS-CoV-2 trimeric spike glycoprotein conformation, its S1(/S2) subunit, or its RBD;
2. Use methods that generate quantitative measures;
3. Always use the same method for the longitudinal monitoring of anti-SARS-CoV-2 antibodies’ kinetics;
4. Prefer laboratory-based assessment.

**Table 4:** Potential advantages and drawbacks of serological testing in recipients of COVID-19 vaccinations.

Advantages	Drawbacks
Assessment of baseline seroprevalence of SARS-CoV-2 infection in non-vaccinated individuals Identification of low- or non-responders to COVID-19 vaccinations Timely detection of the fast decay of anti-SARS-CoV-2 antibody levels	No equivalence to date between anti-SARS-CoV-2 antibody titer and vaccine efficiency No perfect equivalence between anti-SARS-CoV-2 antibody titer and neutralizing activity Cost-effectiveness of different testing strategies needs to be proven Enormous volume of blood drawings and increase of laboratory workload
